# Hyperspectral Imaging for Identification of an Invasive Plant *Mikania micrantha* Kunth

**DOI:** 10.3389/fpls.2021.626516

**Published:** 2021-04-30

**Authors:** Yiqi Huang, Jie Li, Rui Yang, Fukuan Wang, Yanzhou Li, Shuo Zhang, Fanghao Wan, Xi Qiao, Wanqiang Qian

**Affiliations:** ^1^College of Mechanical Engineering, Guangxi University, Nanning, China; ^2^Lingnan Guangdong Laboratory of Modern Agriculture, Genome Analysis Laboratory of the Ministry of Agriculture and Rural Area, Agricultural Genomics Institute at Shenzhen, Chinese Academy of Agricultural Sciences, Shenzhen, China; ^3^College of Mechanical and Electronic Engineering, Northwest A&F University, Yangling, China; ^4^Guangzhou Key Laboratory of Agricultural Products Quality & Safety Traceability Information Technology, Zhongkai University of Agriculture and Engineering, Guangzhou, China

**Keywords:** hyperspectral analysis, invasive plant, data preprocessing, dimension reduction, classification

## Abstract

Mile-a-minute weed (*Mikania micrantha* Kunth) is considered as one of top 100 most dangerous invasive species in the world. A fast and accurate detection technology will be needed to identify *M. micrantha*. It will help to mitigate the extensive ecologic and economic damage on our ecosystems caused by this alien plant. Hyperspectral technology fulfills the above requirement. However, when working with hyperspectral images, preprocessing, dimension reduction, and classifier are fundamental to achieving reliable recognition accuracy and efficiency. The spectral data of *M. micrantha* were collected using hyperspectral imaging in the spectral range of 450–998 nm. A different combination of preprocessing methods, principal component analysis (for dimension reduction), and three classifiers were used to analyze the collected hyperspectral images. The results showed that a combination of Savitzky-Golay (SG) smoothing, principal component analysis (PCA), and random forest (RF) achieved an accuracy (A) of 88.71%, an average accuracy (AA) of 88.68%, and a Kappa of 0.7740 with an execution time of 9.647 ms. In contrast, the combination of SG, PCA and a support vector machine (SVM) resulted in a weaker performance in terms of A (84.68%), AA(84.66%), and Kappa (0.6934), but with less execution time (1.318 ms). According to the requirements for specific identification accuracy and time cost, SG-PCA-RF and SG-PCA-SVM might represent two promising methods for recognizing *M. micrantha* in the wild.

## Introduction

*Mikania micrantha* Kunth (*M. micrantha*), also known as “mile-a-minute,” is one of the world’s 100 most dangerous invasive species (Khadka, 2017). It is estimated that *M. micrantha* can produced between 90,000 and 210,000 seeds/m^2^ (Macanawai et al., 2012; Day et al., 2016). The seeds are dispersed by wind, animals, and humans (Yang et al., 2005; Day et al., 2016). In China, *M. micrantha* achieved an average growth rate of 6–7 cm/day (Zhang et al., 2004; Day et al., 2016). The ecological environment has been seriously damaged, the biodiversity has been threatened, and the economy has been influenced by this weed (Shen et al., 2017). The yield losses of banana (*Musa* spp.), *Citrus* spp., and sugarcane (*Saccharum officinarum* L.) infested with *M. micrantha* ranged from 60 to 70% due to the twining which would block out sunlight (Shen et al., 2013). The economic losses were estimated at US$650,000–1.6 M/year on Neilingding Island (about 554 ha; Zhong et al., 2004). Therefore, identifying and monitoring *M. micrantha* are urgent, which would allow the plant to be controlled by providing accurate information about its geographical distribution (Tesfamichael et al., 2018).

Currently, monitoring *M. micrantha* mainly relied on manual inspection, which is labor intensive and inefficient (Day et al., 2012; Nath et al., 2019). Hyperspectral remote sensing is an efficient monitoring method that has been successfully used to monitor many alien invasive plants (Calvino-Cancela et al., 2014; Sabat-Tomala et al., 2020) and has shown great potential (Chance et al., 2016; Marcinkowska-Ochtyra et al., 2018). In these researches, researchers pay attention to analyzing the raw spectral data characteristics of target invasive plants, extracting spectral signature of the plants, and classifying the features of the plants (Masemola et al., 2019). Some methods, such as random forest (RF), support vector machine (SVM), and their improvements, have been applied for the classification of invasive plants and have achieved good results (Aneece and Epstein, 2017; Grosse-Stoltenberg et al., 2018; Tarantino et al., 2019). It is undeniable that the usage of some spectral wavebands from captured spectral wavebands of hyperspectral images may cause the loss of important spectral information. Nevertheless, the usage of full multispectral bands may cause information redundancy and interference. Therefore, it is necessary to find the balance between them by preprocessing the raw spectral data.

Some state-of-the-art spectral preprocessing detection methods have been proposed by researchers. Liu et al. (2019) used different preprocessing methods to extract hyperspectral reflectance characteristics. A Savitzky-Golay (SG) smoothing of the reflectance spectrum was performed, and the first derivative (FD), the second derivative (SD), and reciprocal logarithm transformation were performed on preprocessed reflectance data by multiple scatter correction and standard normal variate (SNV). The preprocessing methods above have enabled the optimal estimation model to gain better stability and higher precision. To effectively eliminate the noise and baseline hyperspectral drifting, Zhou et al. (2019) proposed a combination of FD, SD, and wavelet transform prepossessing on raw spectral data. Their model achieved 98.57% accuracy in prediction set. Yang et al. (2018) explored the effects of different pretreatment methods on the FT-MIR spectra detection of *Panax notoginseng*, where the best preprocessing combination for the collected spectra was a mix of baseline correction, SNV and FD with an 11 point smoothing. The above preprocessing methods were optimized based on the full-band raw spectral data set and retained all the information of the raw spectral data. However, the calculation workload and time will be increased (Xu et al., 2019). Therefore, this type of method needs to reduce the dimensions of feature sets and keeps most of the dataset information (Luo et al., 2019).

Currently, the methods for reducing the dimension of extracted spectral data from hyperspectral images mainly include feature extraction based on transformation (Du et al., 2018) [e.g., principal component analysis (PCA)] and feature selection based on non-transformation (Salimi et al., 2018; e.g., algorithms for selecting local feature bands). Peerbhay et al. (2015) used hyperspectral remote sensing for the detection and mapping of *Solanum mauritianum* located within commercial forestry ecosystems. This method, based on an RF and PCA, achieved a detection rate of 95% with a false positive rate of 6.39%. Orrillo et al. (2019) used PCA and a classification model preprocessed by an SNV and an SD to identify black pepper adulterated with common adulterant papaya seeds in near-infrared hyperspectral imaging and achieved 100% accuracy in the classification of berry samples. Aneece and Epstein (Aneece and Epstein, 2015) used PCA processed raw spectral data to distinguish among invasive-dominated successional plant communities in the wild. It indicates that different plant species could be identified using spectral information. The previous studies suggest that PCA has been effectively used to reduce raw spectral data dimension, thereby significantly increasing efficiency.

The literature review shows that hyperspectral identification is a potential method for accurate monitoring of *M. micrantha.* Generally, the level of hyperspectral identification can be generally improved only if preprocessing, the feature dimension reduction technique, and the classifier are all addressed (Qiao et al., 2018). Moreover, challenges are manifested in the variability of the raw spectral data of *M. micrantha* in a complex field environment, the lack of prior knowledge and background interference. To address these challenges, hyperspectral preprocessing algorithms [such as FD, SD, nine-point (9P) smoothing, SG smoothing, and SNV], a feature selection algorithm (PCA), and classification algorithms [such as RF, SVM, back propagation neural network (BPNN)] (Vetrekar et al., 2015; Qi et al., 2017) have been proposed, in combination, to recognize *M. micrantha* in wild environments, and an accurate and fast method will be chosen.

## Materials and Methods

### Sample Preparation

A high-speed imaging spectrograph S185 manufactured by the German company Cubert was used to manually collect the *M. micrantha* hyperspectral images in the wild. The spectrometer weighs 470 g, uses DC12V power, and can obtain 138 spectral wavebands with a 4-nm sampling interval in the spectrum range of 450 to 998 nm. The collection site was a desolate field of farmland near the Xinnan subway station in Jiulong town, Guangzhou city, China (23°22′29.5′′ north latitude and 13°29′52.9′′ east longitude). The collection time was approximately 9:30 on November 21, 2018; the weather was cloudy. Before image acquisition dark reference (by closing the camera lens) and white reference (using a white plate) images were collected to calibrate the spectrometer according to the following equation:

(1) $$I_{C}=\left(I_{R}-I_{D}\right)/\left(I_{W}-I_{D}\right)$$

where *I*_*C*_ is the calibrated image, *I*_*R*_ is the raw image, *I*_*W*_ is the white reference, and *I*_*D*_ is the dark reference.

**FIGURE 1:**
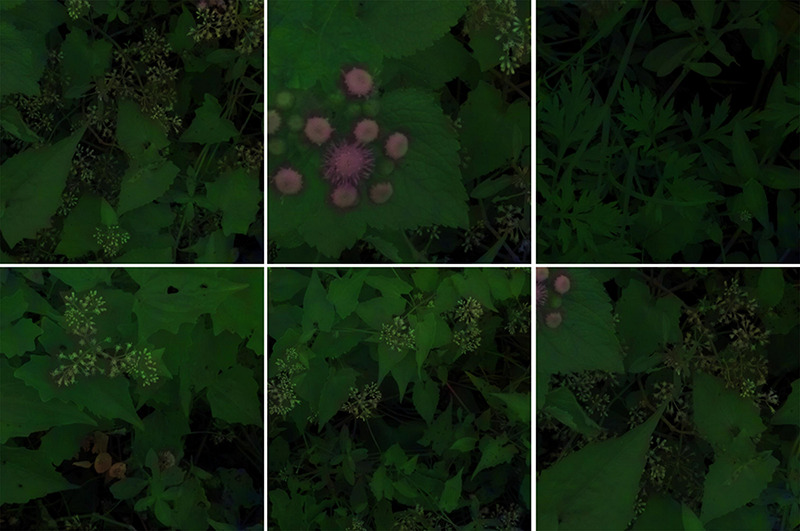
Representative hyperspectral images of *Mikania micrantha* and background.

Then the lens of spectrograph were pointed directly toward at the surface of the plant, and manually focused on the middle of *M. micrantha* leaves. Eighteen hyperspectral images were collected over vegetation using the S185 spectrometer and used for this work. Six samples of the eighteen hyperspectral images contained the leaves and flowers of *M. micrantha*, other plants, and non-plant background are shown in Figure 1. An individual scan time was very short (less than 1 min), and all scans were basically carried out in an area of about 300 square meters. Therefore, it was made within half an hour, and illumination changes from scan to scan varied little.

**FIGURE 2:**
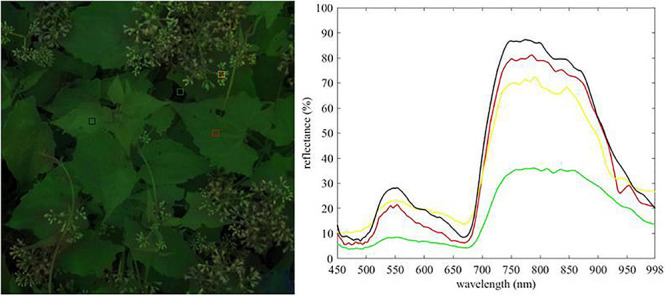
Extraction of raw spectral data. **(A)** Pixels of hyperspectral images used for raw spectra extraction, **(B)** raw spectral data.

The raw spectral data of *M. micrantha* and background were manually extracted from hyperspectral images by Cubeware software (Figures 2A,B), care was taken to avoid any cross-class contamination, and saved in ASCII format. 745 raw spectral data samples (*M. micrantha*: 377, background: 368) were collected and randomly divided into a training set (*M. micrantha*: 251, background: 245), a testing set (*M. micrantha*: 63, background: 62), and a validation set (*M. micrantha*: 63, background: 61). The labels of *M. micrantha* consisted of *M. micrantha* leaves and flowers, and the labels of background included leaves and flowers of other plants, as well as non-plant background. The training, testing, and validation sets were balanced to prevent bias in the classifiers and metrics.

### Methods

**FIGURE 3:**
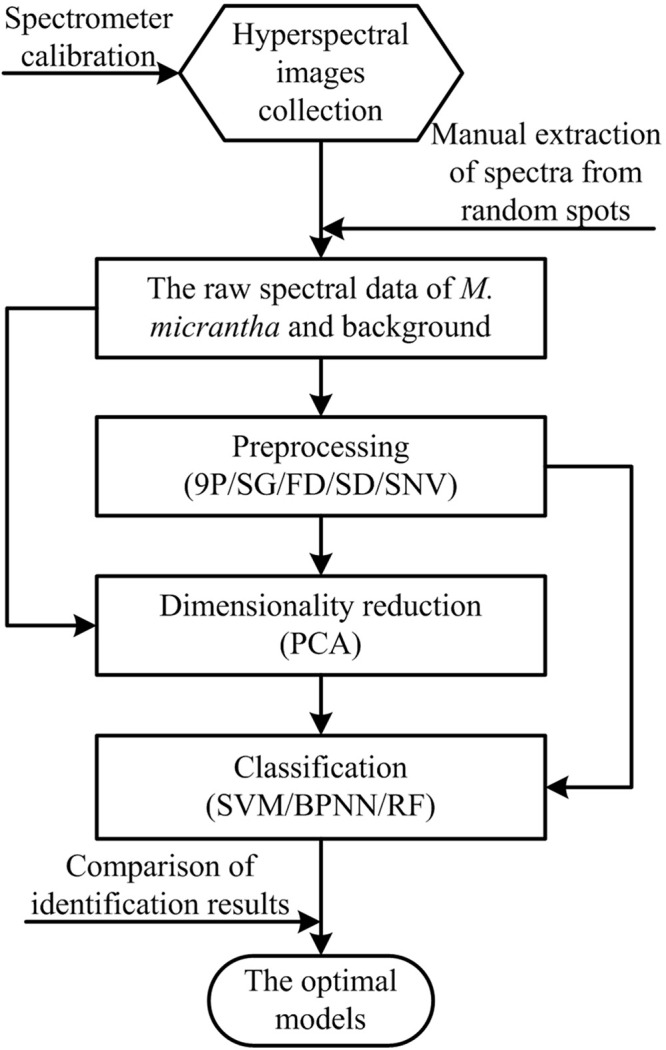
Framework of the proposed methods implementation.

Five preprocessing methods, one feature selection method and three classifiers were combined and implemented to process and classify extracted raw spectral data, respectively. The framework of the proposed methods to recognize *M. micrantha* and choose the optimal model is illustrated in Figure 3.

#### Preprocessing

Smoothing is widely used to eliminate the interference of high-frequency noise in raw spectral data and to improve the spectral signal-to-noise ratio (Saberioon et al., 2019). In this study, 9P smoothing, and SG smoothing were used to smooth the raw spectral data. 9P smoothing can reduce the noise by calculating the average value of a set of sample raw spectral data in the moving smoothing window. The smoothing procedure is as follows. First, the window size was determined to be nine in this article (Lawrence et al., 2006). Second, nine consecutive points on the raw spectral data (*x*_−4_,⋯,*x*_−1_,*x*_0_, *x*_1_,⋯,*x*_4_) were selected. Then, the arithmetic mean was computed and assigned to *x*_*0*_. Finally, the window was moved to the next point so that the center of the window traverses the whole raw spectral data. Similar to 9P smoothing, SG smoothing is a filtering method based on least squares polynomial fitting in a moving window. The window size was set as five in this article (Fu et al., 2018; Liu et al., 2019).

The derivative processing can correct the data far away from the zero plane (Saberioon et al., 2019), thus effectively reducing noise interference, suppressing useless information and highlighting the information of interest. This method is a good choice to deal with noise interference in raw spectral data. First derivative and SD are selected to preprocess extracted raw spectral data. The FD is sensitive to noise and can show the change of reflectivity (Golhani et al., 2019). The SD highlights subtle variations in the spectrum and is suitable for optimal wavelength selection (Wu et al., 2018).

The performance of SNV processing was tested on raw spectral data of *M. micrantha*. SNV assumes the reflection values of each wavelength in the raw spectral data to meet a certain distribution (Yang et al., 2018; Liu et al., 2019), thereby eliminating the errors caused by particle size difference between samples, spectral transformation and surface scattering (Asaari et al., 2018).

#### Dimension Reduction

Raw and preprocessed spectral data have 138 wavebands that makes the feature sets high-dimensional. If the set is directly used for *M. micrantha* target recognition, the calculation workload and time will be increased. Therefore, there was a need to reduce the dimension of the feature set and to keep most of the dataset information. PCA is a transform-based feature extraction method. In this work, PCA was used to transform the raw and preprocessed spectral data, the original high-dimensional raw and preprocessed spectral data were transformed into new comprehensive variable data, while keeping most of the information from the original spectral data (Jeyakumar and Sudha, 2019; Tian et al., 2020).

#### Classification

In the case of limited training samples, the robustness of SVM and RF in processing high-dimensional data makes them suitable for raw and preprocessed hyperspectral data (Tusa et al., 2020). SVM transforms low-dimensional linear inseparable samples into a high-dimensional feature space to make them linearly separable. Based on structural risk minimization, the optimal classification hyperplane is constructed in the feature space to obtain the global optimal solution (Cortes and Vapnik, 1995). RF begins by generating many trees and then votes for the most popular class. This method is an effective tool for classification because each tree depends on the values of a random vector sampled independently and with the same distribution for all trees in the forest (Breiman, 2001).

A BPNN is a multi-layer feedforward network trained by error back propagation. The network takes the sum of error squares as the objective function, and the minimum value of the objective function is calculated by gradient descent method. The commonly used BPNN contains an input layer, an implicit layer, and an output layer. When sufficient training samples are available, the trained BPNN can identify complex objects with high accuracy (Vetrekar et al., 2015; Yao et al., 2019).

To choose the optimal model which is accurate and fast to identify the *M. micrantha* developed from spectral data of hyperspectral image, the three classifiers were tested via different combinations of PCA dimension reduction and other preprocessing methods.

### Evaluation

To evaluate the performance of each of the proposed methods, four statistical parameters, namely, accuracy (A), average accuracy (AA), the Kappa value (Dash et al., 2019) and time, were considered. These parameters are frequently used for performance evaluation in classification problems (Xu et al., 2019). The parameters were calculated from below equations (2), (3), (4), and (5):

(2) Accuracy=(TP+TN)/(TP+TN+FP+FN)

(3) AverageAccuracy=TP/[2(TN+FP)]+TN/[2(TN+FP)]

(4) $$\mathrm{Kappa}=\left(\text{Accuracy}-P_{e}\right)/\left(1-P_{e}\right)$$

$$P_{e}=\left[\left(\text{TP}+\text{FN}\right)\left(\text{TP}+\text{FP}\right)+\left(\text{TN}+\text{FP}\right)\left(\text{TN}+\text{FN}\right)\right]/$$

(5) $${\left(\text{TP}+\text{TN}+\text{FP}+\text{FN}\right)}^{2}$$

where TP is the number of samples correctly predicted to be *M. micrantha*, TN is the number of samples correctly predicted as the background, FP is the number of background samples incorrectly predicted as *M. micrantha*, and FN is number of *M. micrantha* samples incorrectly predicted to be the background.

Also, we summarized the computational time required by each method to recognize the samples in the validation set. All the aforementioned methods were coded and developed in MATLAB R2019a (The Math Works Inc., United States). The CPU of the PC is Intel(R) Core(TM) i7-7700, and the RAM is 16 GB.

## Results

### Preprocessing

**FIGURE 4:**
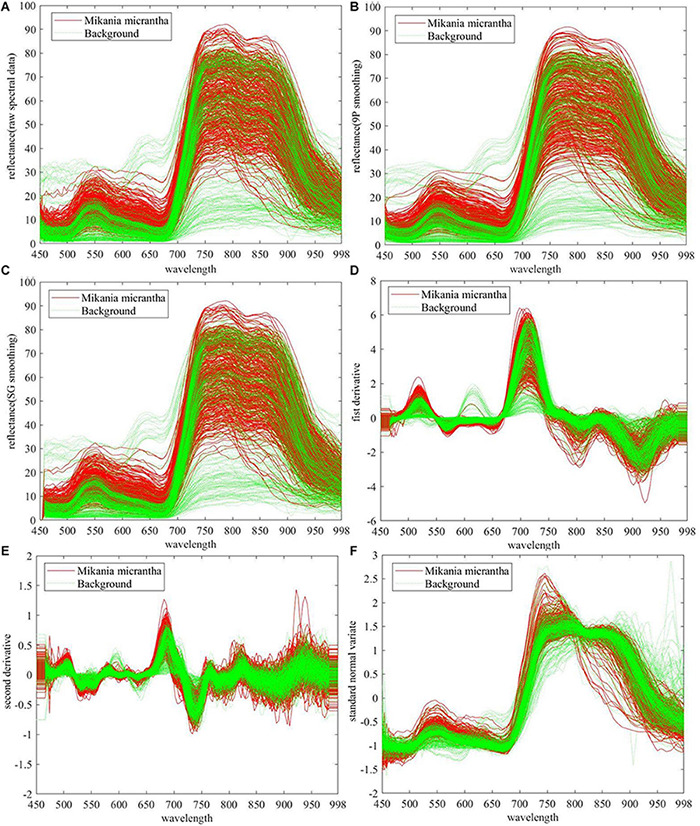
Raw and preprocessed spectral data of 745 samples **(A)** raw spectral data, preprocessed by **(B)** 9P smoothing, **(C)** SG smoothing, **(D)** first derivative, **(E)** second derivative, and **(F)** standard normal variate.

Raw and preprocessed spectral data of *M. micrantha* and background were presented in Figures 4A–F. In Figure 4A, *M. micrantha* spectral reflectance is slightly higher than the background in about 450–670 nm range, while parts of *M. micrantha* have the same reflectance as the background. In about 750–880 nm range, the reflectance of *M. micrantha* and background are scattered, and the reflectance distribution of the background basically overlapped with that of *M. micrantha*. The raw spectral data distribution of *M. micrantha* in the remaining spectral range is almost the same as the background. It indicates that the intra-class differences were more than inter-class differences of *M. micrantha* and background, and it is a challenging work for *M. micrantha* identification. In order to be more conducive to the recognition of raw spectral data, five kinds of preprocessing methods were used to eliminate data noise or highlight the distribution law of reflectance with wavelength. The raw spectral data preprocessed using the two smoothing methods are shown in Figures 4B,C. Compared with the raw spectral data in Figure 4A, the small fluctuations of reflectance over the entire wavelength range (e.g., 450–500 nm) are eliminated or changed more gently. The other three preprocessing methods remove other noises from raw spectral data. The direct analysis of the raw spectral data after derivation is illustrated in Figures 4D,E. The FD and SD were constant states (horizontal line) at both ends of the spectral band (450–470 nm and 978–998 nm), the relevant raw spectral data were obviously polluted by the system noise. In the other spectral range, the intra-class differences of the preprocessed spectral data were smaller than the raw spectral data. Figure 4F shows the preprocessed data of SNV. The intra-class differences of the preprocessed spectral data became smaller, especially the preprocessed spectral data in the 670–880 nm range. And the spectral noise was relatively reduced, too.

In summary, all the five preprocessing methods can eliminate part of the spectral noise. FD, SD, and SVN can significantly reduce the intra-class differences, however, the inter-class differences were not significantly improved by all pretreatments. Therefore, it is necessary to find the difference in the raw and preprocessed spectral data between *M. micrantha* and the background through subsequent processing. To determine the most suitable preprocessing method, the next step was to analyze the influence of each preprocessing method combining the dimension reduction and classifiers on the performance of *M. micrantha* identification.

### Dimension Reduction by PCA

**FIGURE 5:**
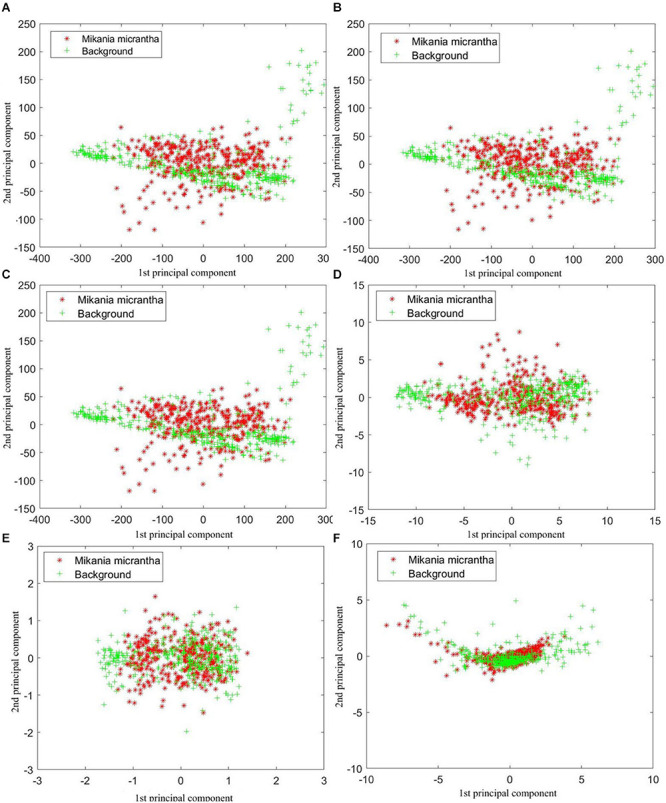
First and second principal component scores of 745 samples based on **(A)** raw spectral data, preprocessed by **(B)** 9P smoothing, **(C)** SG smoothing, **(D)** first derivative, **(E)** second derivative, and **(F)** standard normal variate.

Principal component analysis was performed on the raw and preprocessed spectral data. In general, the first and second principal components have the maximum variation of the original data. The first and second principal component scores of 745 samples were depicted as Figure 5.

Each of the datasets was clustered and distributed with the origin of the coordinates as the center. Figure 5 shows the impact of each preprocessing method on sample clustering. Through comparison of the raw spectral data (Figure 5A) with the raw spectral data preprocessed by different methods, we found that the raw spectral data preprocessed by the SD (Figure 5E) showed the best clustering effect. In terms of clustering performance, the SNV (Figure 5F) ranked second, and the FD (Figure 5D) ranked third. Compared with the degree of clustering of the raw spectral data (Figure 5A), the clustering effect of the two smoothing treatments was the worst (Figures 5B,C). Nevertheless, the above results are predictable because the two smoothing treatments reduced the noise of the raw spectral data but did not change the details and the overall trend. In addition, the overlap between the two types of samples was obvious as shown in Figure 5. Thus, more principal components need to be taken into account.

**FIGURE 6:**
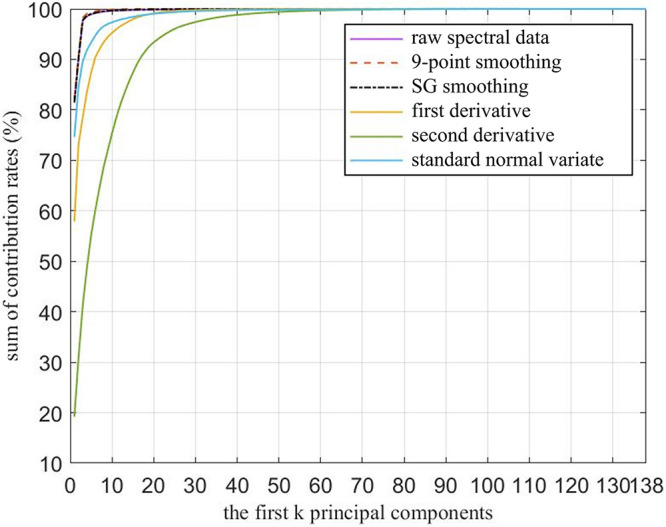
Cumulative contribution rate curve of the principal components.

As shown in Figure 6, the cumulative contribution rates of the first *k* (*k* = 1, 2,…, 138) principal components were also calculated. The raw spectral data, 9P smoothing, and SG smoothing had almost the same curves, and at approximately the first 5 principal components, all curves tended to be smooth and close to 100%. Thus, all the curves are able to fully represent the information contained in the 138-dimensional raw and preprocessed spectral data. Although the contribution rate of the first principal component of the FD was approximately 20% lower than that of the SNV, the FD, and SNV did not have almost the same cumulative contribution rates until the first 18 principal components, where the values are close to 99%. The SD had the lowest contribution rate of the first principal component, and the cumulative contribution rates were not more than 99% until top 40 principal components. Therefore, the first *k* principal components based on different preprocessing contained most of the information. To achieve accurate *M. micrantha* identification, the first *k* principal components were selected as the input of the classification model. However, the *k* values were dependent on the preprocessing and classification algorithms combined with PCA.

### Recognition Performance Assessment Based on Different Combinatorial Algorithms

**FIGURE 7:**
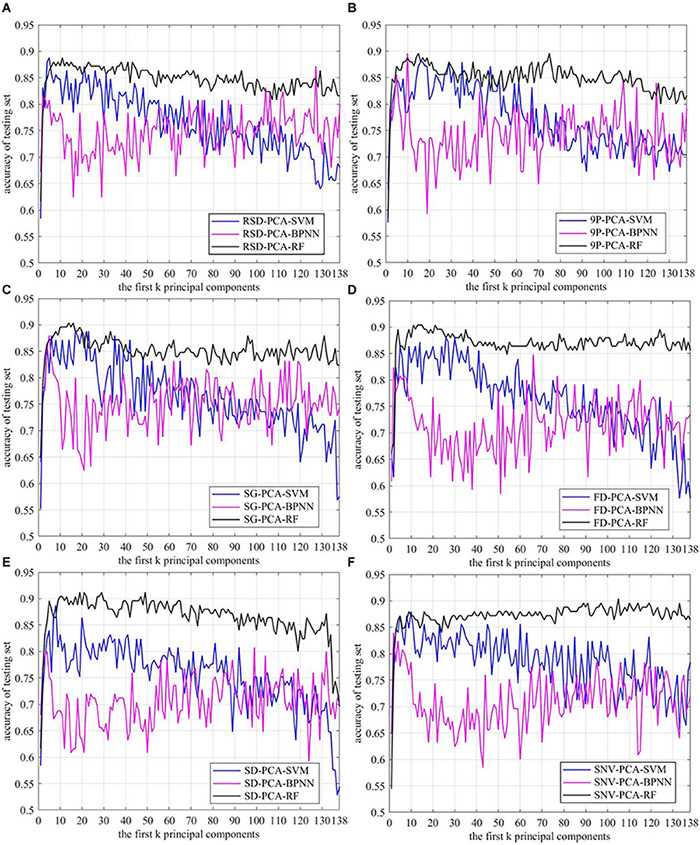
Recognition accuracy curve of *Mikania micrantha* based on the first *k* principal components. **(A)** raw spectral data, preprocessed by **(B)** 9P smoothing, **(C)** SG smoothing, **(D)** first derivative, **(E)** second derivative, and **(F)** standard normal variate.

Among the 745 samples, 496 samples were used to train the models, and 125 samples were used to test the trained models. The SVM, the BPNN, and the RF were separately trained based on the first *k* (*k* = 1, 2,…, 138) principal components, which were generated by employing PCA on raw and preprocessed spectral data. The recognition accuracies of the first *k* principal components are shown in Figure 7.

The results showed that the accuracy of the RF was significantly higher than the other two methods. When *k* > 10, the accuracies of the adjacent first *k* principal components fluctuated within the smallest range. The SVM was the second most accurate; its accuracy first increased and then decreased with the increase of *k*. The BPNN did not achieve good results, and the accuracies of adjacent *k* fluctuated within a large range, although the accuracy was higher than that of the SVM as the *k* increased to a certain degree. Using the same classification method, the accuracies of SG-PCA-RF, FD-PCA-RF, and SD-PCA-RF methods were more significantly improved than the accuracy of OR-PCA-RF, but 9P-PCA-RF did not achieve much improvement except for when the first *k* principal component was between 70 and 80. The accuracy of SNV-PCA-RF improved as the *k* increased, but there was little benefit in terms of dimension reduction. In Figures 7C–E, the maximum accuracy of the RF appeared at *k* between 10 and 20, and the accuracies of SD-PCA-RF were higher than those of SG-PCA-RF and FD-PCA-RF. In addition, compared with OR-PCA-SVM, the combinations of the other preprocessing methods with PCA and SVM did not improve accuracy obviously. And the *k* values corresponding to the maximum accuracy of the other combination methods were higher than that of OR-PCA-SVM, such as 9P-PCA-SVM, SG-PCA-SVM, and FD-PCA-SVM. For the BPNN algorithm, 9P smoothing and SG smoothing improved the accuracy and reduced the dimensions, while the *k* values corresponding to the maximum accuracy were located at between 1 and 10. However, the accuracy of other preprocessing methods was lower than the raw, thereby having even negative effects.

Therefore, not all combinations of preprocessing methods, PCA and classification methods improved accuracy while reducing the dimensions. To reduce the dimensions and improve accuracy, the first *k* principal components corresponding to the maximum accuracy of each combination method were separately confirmed to reduce the dimensions (Table 1) and to verify the recognition performance.

**TABLE 1:** First *k* principal components corresponding to the maximum accuracy of each combination method.

***k*/max accuracy (%)**	**RSD**	**9P smoothing**	**SG smoothing**	**FD**	**SD**	**SNV**
PCA-SVM	5/88.80	16/88.00	17/88.80	26/88.00	8/88.80	10/88.00
PCA-BPNN	127/87.20	10/89.60	5/88.00	66/84.80	99/80.80	2/84.00
PCA-RF	11/88.80	15/89.60	14/90.40	11/90.40	19/91.20	105/90.40

*RSD is raw spectral data.*

The remaining 124 samples were used as the validation set to verify the corresponding trained models at the first *k* principal components as shown in Table 1. There were over 10 runs for each method. The most frequent results are shown in Table 2. The best results for each quality index were highlighted in bold. The result shows that SG-PCA-RF yielded the best A, AA, and Kappa values, and 9P-PCA-SVM had the shortest execution time among all the methods (Table 2).

**TABLE 2:** Methods combining preprocessing, PCA, and a classifier for validation set recognition.

**Methods**	***k***	**Validation set**			
		**A (%)**	**AA (%)**	**Kappa**	**Time (ms)**
RSD-PCA-SVM	5	81.45	81.62	0.6302	0.963
RSD-PCA-BPNN	127	78.23	78.13	0.5636	6.169
RSD-PCA-RF	11	83.87	83.84	0.6772	10.275
9P-PCA-SVM	16	83.87	83.81	0.6770	**0.823**
9P-PCA-BPNN	10	75.81	75.77	0.5158	6.082
9P-PCA-RF	15	84.68	84.63	0.6932	10.028
SG-PCA-SVM	17	84.68	84.66	0.6934	1.318
SG-PCA-BPNN	5	81.45	81.46	0.6290	5.575
SG-PCA-RF	14	**88.71**	**88.68**	**0.7740**	9.647
FD-PCA-SVM	26	80.65	80.69	0.6132	1.115
FD-PCA-BPNN	66	70.97	70.99	0.4195	5.332
FD-PCA-RF	11	83.87	83.89	0.6775	10.571
SD-PCA-SVM	8	79.84	79.98	0.5978	1.014
SD-PCA-BPNN	99	72.58	72.50	0.4506	6.033
SD-PCA-RF	19	86.29	86.20	0.7252	10.653
SNV-PCA-SVM	10	81.45	81.49	0.6292	1.305
SNV-PCA-BPNN	2	82.26	82.31	0.6454	5.533
SNV-PCA-RF	105	85.48	85.51	0.7098	10.431

*RSD is raw spectral data. The best results for each quality index were highlighted in bold.*

## Discussion

In the process of exploring the identification of *M. micrantha* based on hyperspectral technology, the combinatorial test of conventional spectral data processing methods was carried out. The results showed that RF and SVM based on homologous preprocessing spectral data maintained the advantages of accuracy and time, respectively. In terms of the recognition effect of *M. micrantha*, RF shows higher accuracy and recognition consistency than the other two classifiers. Certainly, if the time indicator is the most important in practical applications, SVM is also a good choice. After all, it also has a satisfactory accuracy and consistency.

When applied to the same classifier RF, SG smoothing yielded the best A, AA, Kappa, and time values, and SD yielded the second best A, AA, and Kappa values. When applied to SVM, SG smoothing yielded the best A, AA, and Kappa values, and 9P smoothing yielded the second best A, AA, Kappa values, and best time, but the improvement was not obvious compared with OR. The above results were basically consistent with the analysis results in Figure 7 and Table 1. Overall, SG smoothing worked the best among the five common pretreatments tested during *M. micrantha* identification using hyperspectral image data. Moreover, the methods combining preprocessing with a classifier were also used for validation set recognition, without PCA. The results are shown in Table 3. Compared with Table 2, all indexes were worse in most of the cases as shown in Table 3. Although SNV-RF was better than SNV-PCA-RF in terms of A, AA, and Kappa, SNV-RF was still inferior to SG-PCA-RF in all indexes. Even for the BPNN classifier, which showed the worst comprehensive performance in *M. micrantha* recognition, PCA dimension reduction treatment can improve the recognition effect. However, the recognition effect fluctuated significantly with the change of the number of principal components. Therefore, PCA was able to improve the accuracy and efficiency of the algorithms in most cases.

**TABLE 3:** Methods combining preprocessing with a classifier for validation set recognition.

**Methods**	**Validation set**			
	**A (%)**	**AA (%)**	**Kappa**	**Time (s)**
RSD-SVM	81.45	81.38	0.6285	**1.823**
RSD-BPNN	66.94	66.94	0.3387	5.963
RSD-RF	83.06	82.92	0.6602	12.665
9P-SVM	82.26	82.18	0.6445	3.146
9P-BPNN	71.77	71.86	0.4364	5.857
9P-RF	83.87	83.81	0.6770	9.616
SG-SVM	83.37	83.79	0.6768	1.900
SG-BPNN	71.77	71.86	0.4364	6.795
SG-RF	84.68	84.56	0.6927	9.350
FD-SVM	83.06	83.02	0.6609	2.049
FD-BPNN	77.42	77.39	0.5480	30.801
FD-RF	85.48	85.48	0.7096	9.329
SD-SVM	81.45	81.49	0.6292	21.212
SD-BPNN	80.65	80.64	0.6128	10.110
SD-RF	86.29	86.22	0.7254	10.605
SNV-SVM	82.26	82.33	0.6456	11.373
SNV-BPNN	71.77	71.78	0.4355	17.394
SNV-RF	**87.10**	**87.12**	**0.7420**	12.048

*RSD is raw spectral data. The best results for each quality index were highlighted in bold.*

In summary, the SG-PCA-RF (88.71% A, 88.68% AA, 0.7740 Kappa, and execution time of 9.647 ms) and SG-PCA-SVM (84.68% A, 84.66% AA, 0.6934 Kappa, and execution time of 1.318 ms) algorithms outperformed other methods for *M. micrantha* recognition. Therefore, the method should be selected according to the specific requirement for identification accuracy and time cost.

The recognition methods based on convolutional neural network (CNN) are very popular at present, however, it does not mean that these methods are applicable to all researches. Fernandes et al. (2019) used SVM and CNN to identify the hyperspectral image data of different grape vine varieties, and the test results showed that SVM achieved a recognition effect not inferior to CNN. Of course, we recognize that deep learning is a trend of image recognition. In order to further improve the recognition accuracy and consistency, it is necessary to expand the training set and employ the recognition method based on deep learning.

The main work of this research was to complete the identification of *M. micrantha* in a small field. The image samples used were hyperspectral images taken with a handheld spectrometer. In the future research, the hyperspectral images of other invasive plants will be collected to verify the generalization performance of the proposed method. In addition, the images acquired by the handheld spectrometer were mainly used to study the hyperspectral image data processing method, which can save time and cost on the basis of ensuring the reliability of the data. In practical applications, it is often necessary to identify invasive plants in a wide range, which requires the hyperspectral imager to be mounted on the UAV for image acquisition. Our study provides a reliable reference for hyperspectral image data processing of *M. micrantha*.

## Conclusion

In this study, to determine the best methods for *M. micrantha* recognition based on hyperspectral technology, five preprocessing methods, one dimension reduction method, and three classifiers were separately combined to process the hyperspectral image data of *M. micrantha*. It was demonstrated that SG smoothing could eliminate the interference of high-frequency noise in raw spectral data and improved the spectral signal-to-noise ratio. Importantly, PCA reduced the dimensions of the feature set and kept most of the dataset information. Additionally, PCA improved the accuracy and calculation efficiency of the algorithm to some extent. In our study, the recognition accuracy and time after PCA dimension reduction were universally better than those without PCA processing. Finally, the dataset after dimension reduction was classified by classifiers, proving that RF had the most accurate and consistent result in our dataset, while SVM had the shortest execution time. In subsequent studies, SG-PCA-RF and SG-PCA-SVM algorithms, which performed well in this study, will be tested in the hyperspectral images of other invasive plants obtained by UAV.

## Data Availability Statement

The raw data supporting the conclusions of this article will be made available by the authors, without undue reservation.

## Author Contributions

YH and XQ: methodology, software, validation, writing – original draft, and writing – review and editing. JL: methodology, software, and validation. RY: methodology and software. FuW: software. YL and SZ: project administration. FaW: writing – review and editing. WQ: project administration, writing – original draft, and writing – review and editing. All authors contributed to the article and approved the submitted version.

## Conflict of Interest

The authors declare that the research was conducted in the absence of any commercial or financial relationships that could be construed as a potential conflict of interest.
